# Editorial Comment: ^68^Ga-Prostate-specific membrane antigen (PSMA) positron emission tomography (pet) in prostate cancer: a systematic review and meta-analysis

**DOI:** 10.1590/S1677-5538.IBJU.2019.0817.1

**Published:** 2021-07-20

**Authors:** Stênio de C. Zequi

**Affiliations:** 1 A. C. Camargo Cancer Center Fundação Prudente São PauloSP Brasil A. C. Camargo Cancer Center, Fundação Prudente, São Paulo, SP, Brasil

## COMMENT

Matushita and colleagues performed a comprehensive review and meta-analysis about the role of ^68^Ga PSMA PET in the diagnostic and in re staging of prostate cancer based on the final selection of 35 studies with more than 3900 patients in this issue publication: Ga-Prostate-specific membrane antigen (psma) positron emission tomography (pet) in prostate cancer: a systematic review and meta-analysis (1). The evaluated series were heterogeneous, since the review encompassed, as patients submitted to prostate biopsy (diagnostic), as patients underwent radical prostatectomy, radiotherapy or lymphadenectomy, and some series including MRI in combination with ^68^GA PSMA PET, also.

The use of ASTRO 1996 definition in this review, seems at first sight an interesting choice, since the lower PSA cut-off for relapse (three consecutives PSA elevations >0.2 ng/mL), when compared with Phoenix Definition (nadir plus 2.0 ng/mL), could result, in early anatomic diagnostics of the recurrence sites by this nuclear scan, which could result in early precise salvage treatments. However, an ASTRO consensus, in 2006, has recommend by the limitation use of ASTRO definition only for patients undergone exclusive external beam radiotherapy, since this failure definition perform poorly in patients which received hormonal therapy (2).

The review manuscript corroborated the high sensitivity and positivity from ^68^ PSMA at diagnostic. It is really interesting mainly in when focal therapy is planned, being as tool option for exclusion of some non-diagnosticated contralateral lesion after an anatomopathological test revealing only unilateral cancer.

On the other hand, for bilateral tumors, evolving the whole gland, in the era of fusion biopsy, probably ^68^Ga PSMA PET might be less ordered, because in this moment, anatomopathological tests must not be excluded or replaced by functional image methods. In this scenario, perhaps patients with high suspicion for prostate malignancies with previous negative biopsies, can be benefited by the use of ^68^Ga PSMA (combined or not with MRI).

A great daily clinical practice challenge, is the re-staging of recurrent prostate cancer, with was well discussed in the paper. We must reinforce that authors shown that in the biochemical recurrence studies, a quarter of cases, the ^68^Ga PET CT are negatives. Although it could sound unfavorable, in a recent study from our group (3), which evaluated biochemical recurrence after primary treatments, with ^68^Ga PSMA PET, in 57 patients with low and intermediate risks prostate cancer, we verified that in half of them (49.12%) presented negative PET scans; 11 of whom undergone salvage therapies and achieved 90% of significant PSA decline. Among, the remaining (50,8%) PET CT positive patients, the ^68^Ga PSMA PET findings enhanced the discrimination between patients with local recurrences, treated by salvage local radiotherapy from the patients with distant dissemination, better candidates to systemic therapies.

The review text brings a broad overview (until April 2019) of the use of ^68^Ga PSMA PET and its accuracy in the main clinical indications in an area of a great interest of literature in the last few years. The read of this literature syntheses must be recommended as subside for the reader for the future better understanding of functional image tests in prostate cancer: when we search the mesh term “psma pet prostate cancer” in the PUB MED website, we found more than 890 articles published between April 2019 up March 2021. It will be a hard task to be update in the next future.

More news are coming. Rauscher et al, in 2020, demonstrated that ^68^GA PSMA detect five times less benign lesions in comparison with 18F PSMA 1007 (55 versus 245; p<0,001), benign lesions were more frequently found in: ganglia, no specific lymph nodes and in skeleton, in face of these findings, specific image readers’ training might be dedicated according the isotope is used in PSMA in each pet scan modality is used (4).

Although the use of ^68^PSMA PET in the biochemical recurrence can detect pelvic lymph nodes in unusual locations, favoring the planning of salvage radiotherapy, conversely, in the spectrum of salvage lymphadenectomy guided by PSMA ligand PET, there are several open questions nowadays: microscopic spread to adjacent positive lymph nodes can be not detected (5). We are not sure if the resection of sole positive nodes during the salvage lymphadenectomy can be effective. The adequate biochemical control after salvage lymphadenectomy guided by images from PSAM ligand PET, usually are reached only by 19-59% of the patients, and in many of them, only by short time length. More durable results have been verified in cases in which a single lesion is positive. If positivity of PSMA PET in unilateral, is really necessary to remove the contralateral nodes? Must we resect nodes in an anatomical level above or in an anatomical level below the positive PET Scan lesions? Future well controlled series are more than desirable to solve many of these doubts. For better understanding in the future, for sure, this review and meta-analysis from Brazilian and Italian authors, is so helpful.
